# The Influence of Varying Atmospheric and Space Weather Conditions on the Accuracy of Position Determination

**DOI:** 10.3390/s23052814

**Published:** 2023-03-04

**Authors:** Maciej Nowakowski, Ewa Dudek, Adam Rosiński

**Affiliations:** 1 Demant Technology Centre Sp. z o.o., Al. Jana Pawła II 22, 00-133 Warsaw, Poland; 2 Faculty of Transport, Warsaw University of Technology, 75 Koszykowa St., 00-662 Warsaw, Poland; 3 Division of Electronic Systems Exploitations, Institute of Electronic Systems, Faculty of Electronics, Military University of Technology, 2 Gen. S. Kaliski St., 00-908 Warsaw, Poland

**Keywords:** satellite systems, navigation, position determination, precise positioning, weather influence

## Abstract

Today’s technological developments make it possible to use machines to perform specific tasks instead of humans. However, the challenge for such autonomous devices is to precisely move and navigate in constantly changing external environments. In this paper, the influence of varying weather conditions (air temperature, humidity, wind speed, atmospheric pressure, type of satellite systems used/satellites visible, and solar activity) on the accuracy of position determination was analyzed. To reach the receiver, a satellite signal must travel a great distance and pass through all layers of the Earth’s atmosphere, the variability of which causes errors and delays. Moreover, the weather conditions for receiving data from satellites are not always favorable. In order to investigate the impact of delays and errors on position determination, the measurements of the satellite signal were conducted, the motion trajectories were determined, and the standard deviations of these trajectories were compared. The results obtained show that it is possible to achieve high precision in determining the position, but varying conditions, such as solar flares or satellites’ visibility, meant that not all measurements are able to achieve the required accuracy. The use of the absolute method of satellite signal measurements contributed to this to a large extent. To increase the accuracy of positioning by GNSS systems, it is first of all proposed to use a dual-frequency receiver that eliminates ionospheric refractions.

## 1. Introduction

Today’s technological developments make it possible to use machines to perform specific tasks instead of humans. Machines can replace people in various activities: stationary as well as movable. In this paper, attention is paid to the latter. However, if they move in a constantly changing external environment, it is a challenge to navigate them precisely. The problem analyzed in this research refers to the influence of varying weather conditions on the accuracy of the determination of navigational position. In many applications (such as car driving), navigation positioning accuracy at the level of 5–6 m is sufficient, especially thanks to the driver’s experience. However, there still are other applications, such as autonomic machines for example, in which human senses and intelligence may not be used and as a consequence the positioning must be more precise.

During real-time navigation to reach the receiver, a satellite signal must travel a great distance and pass through all layers of the Earth’s atmosphere, the variability of which causes errors and delays (mainly ionospheric and tropospheric). Moreover, the weather conditions for receiving data from satellites are not always favorable, while navigated machines/robots must operate independently of them. In order to investigate the impact of delays and errors on such a navigation, the measurements of the satellite signal were conducted, the trajectories of the actual and theoretical measurements were determined, and the standard deviations of these trajectories were compared. Conclusions were drawn based on these measurements.

The issue of the impact of changing weather conditions on the accuracy of position determination has been of interest to scientists for many years [1,2,3]. This is due to the fact that in many areas of economy, including, in particular, transport, it is necessary to correctly determine the location of a moving vehicle/companion robot/pedestrian [4,5,6,7] in order to take advantage of the services offered by intelligent transport systems [8,9,10,11,12]. Meanwhile, position determination [13,14,15] is not only affected by failures of global navigation satellite systems (GNSS) receivers [16,17,18,19,20,21,22], but also by weather conditions [23,24], which can have significant influence on position accuracy. Thus, in effect, there may be a threat in terms of the safety of people and vehicle movement [25,26,27].

More credible positional determination can be obtained by minimizing the positioning error. This is possible, e.g., by developing innovative solutions in GNSS receivers. This approach was presented in the article [28]. The authors proposed the use of time-differentiated carrier phase (TDCP) measurements. This is particularly advantageous in urban environments where signal multipathing occurs. Field studies confirmed the legitimacy of this solution, however, it would be beneficial to take into account environmental conditions during the experiments.

Another possibility to improve positioning accuracy is the integration of receivers using different navigation systems. Such an approach was presented in article [29], in which the authors proposed to integrate global positioning system (GPS) and BeiDou receivers, which resulted in improvement in the positional data accuracy and reliability. This was achieved by increasing the number of visible satellites moving in different orbits. In this way, the impact of weather conditions on the accuracy of positional determination was also minimized. A similar approach, but based on statistical techniques, was described in publication [30].

A reduction in the impact of weather conditions on the accuracy of position determination is also possible by increasing the number of systems used. In article [31], the authors used four GNSS systems (i.e., GPS, Глобaльнaя нaвигaционнaя cпyтникoвaя cиcтeмa (GLONASS), BeiDou (北斗卫星导航系统), European Geostationary Navigation Overlay Service (EGNOS)). Conducted analysis shows that the four-constellation positioning system significantly shortens the convergence time. There is also an improvement in positioning accuracy compared to a single GPS system.

Another approach used in scientific studies is the implementation of proprietary software in GNSS receivers. Scientists in this area use position data obtained with other information and communication technologies (ICT) solutions, thanks to which it is possible to increase the correctness, accuracy, and reliability of positioning data [32,33,34]. Such solutions, to a certain extent, make it possible to become independent of the variability of weather conditions.

Position accuracy is affected not only by terrestrial atmospheric conditions [35], but also by solar activity (such as solar storms for example) [36,37,38,39]. Research in this area was presented by the authors in article [40]. To minimize the adverse impact of solar storms on the BeiDou system’s functioning, the number of satellites in this system was increased. In the system expansion analyzed, the authors considered both—to increase the number of satellites by a dozen or so, as well as by up to 60, which significantly reduces the impact of large cosmic observation errors, but is associated with the significant costs of system expansion [41,42].

As shown in the literature review, satellite systems often appear in research papers. There are ways to reduce the impact of various factors on position determination. Some of them are applied in this paper as well. However, attention should be paid to the obstacles that may contribute to all kinds of failures in the functioning of receiver—satellite system pairs. One such obstacle certainly is the constantly changing weather conditions, which greatly affect the navigation. Furthermore, by combining various fields of science, engineering, and technology, it is possible to create and practically use autonomic machines. Moreover, their applications are becoming more and more frequent, for example in a form of robots accompanying elderly people in their everyday activities, so their movements must be precise. In the authors’ opinion, there is a lack of publications concentrating on such an approach. That is why the main aim of this paper is to verify the influence of varying weather conditions on the accuracy of positional determination during navigation. The authors pose the following questions: (a) is the accuracy of all measurements equal?; (b) does it depend on the weather condition?; (c) what can be done to increase the positioning? The structure of the article, allowing the main aim’s achievement, is as follows: background of the research and review of the literature (Section 1), short introduction of satellite systems and errors in position determination (Section 2), satellite signal measurement in various weather conditions (Section 3), and, finally, conclusions and summary (Section 4).

## 2. Satellite Systems and Errors in Position Determination

Satellite systems are an integral part of human civilization nowadays. They allow positioning as well as navigation in real time. Thanks to even the simplest smart device, it is possible to reach a specific destination by the shortest and the fastest route, and this is just the outset.

### 2.1. Satellite Systems Short Introduction

Development of satellite systems began in the 1970s, and, basically, from the beginning of the 21st century they have become widely used. Satellite navigation technologies are used everywhere [43,44,45,46,47,48,49,50,51,52,53]: in archaeology, construction, geodesy, mining, transport, security services, or agriculture. Even devices such as hearing aids have built-in GPS receivers so that the user can find them at the application level. As technology advances at a dizzying pace, it is inevitable that future machines will be able to operate autonomously using satellite technology for location and navigation. The main satellite systems are listed as follows:

- Global positioning system (GPS) arising from the American Navy Navigation Satellite System (NAVSAT), which was initially used for US Navy submarine positioning and was the first to allow satellite navigation;
- European satellite system—Galileo, a relatively young system, launched in 2016, developed in Europe beginning in the 1980s due to the fear of the inaccessibility of the GPS and GLONASS systems;
- Russian positioning system—GLONASS;
- Chinese satellite system—BeiDou.

What all four of them have in common is their method of operation, similar in all satellite systems. Several dozen satellites circulating around the Earth’s orbit send satellite signals to on-earth receivers. Based on the knowledge of the electromagnetic wave propagation speed and the position of at least four satellites, receivers are able to determine their geographical location on the ground, which is the starting point for their “guided movements”. This is where the first question arises—which of the available systems should be used in case of such an approach, based on position acquisition, and its implementation in autonomous machines operating outdoor? The answer is: all of them. Today’s devices have various global navigation satellite systems (GNSS) modules built in. They can observe different circulating satellites from various satellite systems. Such a solution reduces the probability of the event that less than four satellites (the lowest limit number) will be visible to the receiver.

The receiver used for measurements, analyzed in this paper, and described in Section 3, also has the advantage of observing satellites from different satellite systems.

Satellite signal measurements may be carried out in various ways. Depending on the obtained data purpose, different methods are used. In this research, the absolute measurement method was used, although the authors are aware that this is not the most precise available method. However, it requires one GNSS receiver only, so it was decided to analyze such an option to verify the accuracy of positioning in case an autonomous machine is equipped with one receiver only (or at the moment that only one receiver works properly). Is it going to be a restraint with changing weather conditions or not?

### 2.2. Errors in Position Determination

Satellite systems should be treated as advanced technologies. The problem is that the individual components of the system are separated from each other by thousands of kilometers. This complicates the reach of adequate accuracy in position determination. The satellite signal has to travel a very long distance, during which it encounters interference related to Earth’s atmosphere, as well as problems caused by, for example, urban agglomeration (large buildings can block the simplest path to the receiver). All in all, there are many factors affecting the accuracy of satellite systems. In this paper, the greatest attention was paid to errors arising from weather conditions (especially solar flares) and related to the Earth’s atmosphere.

Satellite navigation systems in various applications require high positioning accuracy. Then, appropriate methods should be used to eliminate errors in determining the position, which can reach even several dozen meters. However, first, it is necessary to know what error types are the most common. These are [54]:

- Ephemeris errors—satellite position errors, basically, the difference between their real and identified positions; they are caused by the Earth’s gravitational field, atmospheric drag, the gravitational effects of the Sun, Moon, and other celestial bodies, solar radiation, crustal tides, oceanic tides, electromagnetic forces, and relativistic effects; the error caused by the ephemeris can be about 2 m, but can be reduced;
- Inaccuracy of the time standard—position determination is mainly related to the measurement of the time, in which the signal reaches the receiver from the satellite; since the speed of wave propagation in vacuum is 300,000 km/s, it is important to know the propagation time of the signal, because a small deviation of the time can cause errors of several meters;
- Signal multipath—multipath errors are related to secondary wave inference; the phenomenon occurs when the satellite signal does not reach the receiver directly, but through various paths due to reflections from all kinds of objects standing in the way of the signal; in particular, the phenomenon of multipathing can be seen in large cities, where big buildings are present in high numbers, and when the satellites are low on the horizon;
- Variation of the antenna phase center—this error appears when the physical center is not compatible with the phase center of the receiver’s antenna; the phase center is constantly changing; due to the constant change in the height and azimuth of the satellites, the angle of signal transmission also changes; deviations caused by this phenomenon are generally small and the newer the antenna, the smaller the deviation, up to several millimeters;
- Receiver’s noise—noise is nothing different then a voltage peak of random frequency and amplitude, generated on current-carrying elements; the satellite receiver itself is a source of unwanted noise; noise affects accuracy and cannot be eliminated;
- Geometric errors of the satellite alignment—this type of error is affected by the satellites’ position versus the receiver; the error is described by dilution of precision (DOP)—a parameter characterizing the influence of satellite constellation geometry on positioning; if any of the coefficients are equal to zero, it means that the measurement is impossible due to interference, weak signal from the satellites, or too few visible satellites;
- Errors in the design of the satellite system—satellites’ location has a significant impact on their visibility to the receiver; four visible satellites are indispensable for positioning, however, this is the minimum vital number and may cause positioning errors;
- Errors related to the Earth’s atmosphere (which are discussed hereinafter).

An atmosphere is a gaseous shell that surrounds a celestial body, with enough mass to hold a layer of gases as a result of gravity. In the case of satellite systems, two atmosphere layers have a significant impact on their operation: the troposphere and the ionosphere. The troposphere is the layer of the Earth’s atmosphere closest to the Earth, occupying the smallest area in relation to the other layers, but having the largest mass. It makes up 75% of the total mass of the Earth’s atmosphere and is a place where most weather phenomena occur. The troposphere consists of a mixture of gases, mainly azote and oxygen, as well as industrial gases such as carbon dioxide, sulfur, etc. The troposphere is characterized by a continuous decrease in temperature and pressure with increasing altitude. The average decrease in temperature is 0.6 °C per 100 m, while the average pressure above sea level is 1013 hPa, and about 300 hPa at an altitude of 10 km.

Tropospheric error, influencing satellite systems operation, is caused by the refraction of the electromagnetic wave. The dry part of the troposphere, characterized by atmospheric pressure, is responsible for 90% of the positioning error, while 10% is the wet part, characterized by water vapor. The parameter describing the tropospheric delay of the satellite signal related to atmospheric pressure is zenith hydrostatic delay (ZHD). ZHD depends mainly on the refraction associated with the hydrostatic part of the atmosphere, i.e., with the dry part of the atmosphere, and can be calculated based on deterministic models—the Hopfield model for example [55]:

(1) $$ZHD_{Hop}=2*10^{7}*N_{hyd}*H_{d}$$

where

(2) $$N_{hyd}=k_{1}*\frac{P}{T_{k}}$$

(3) $$H_{d}=40136+148.72*T_{c}$$

$k_{1}$—refraction index, always greater than 1;

*P*—atmospheric pressure;

$T_{K}$—temperature expressed in Kelvins;

$T_{C}$—temperature expressed in degrees Celsius.

The error due to the wet part of the atmosphere is usually neglected. In order to eliminate the tropospheric refraction error, corrections are applied to the measurement in a form of special mathematical models, such as the Saastamoinena model for example [55]:

(4) $$ZWD_{Saas}=0.00227768*\left(\frac{1255}{T_{K}}+0.05\right)*e$$

where

ZWD—zenith wet delay;

$T_{K}$—temperature expressed in degrees Celsius;

e—water vapor pressure at the height of the reference station.

The ionosphere is an ionized layer of the atmosphere, starting at 85 km above the Earth’s surface, reaching an altitude of 2000 km in the exosphere. The essence of the ionosphere is that it contains plasma, which is formed by the ionization of gas particles in the atmosphere by cosmic rays and ultraviolet solar radiation. The ionosphere, through its structure, affects the refraction, reflection, absorption, and polarization of radio waves. These phenomena cause interference in radio communication. That is why positioning errors caused by the ionospheric layer of the Earth’s atmosphere consist of electromagnetic waves’ propagation speed changes when passing through this layer. Wave speed changes arise from ionized gases influence, as a result of solar activity and cosmic radiation, and are dependent on the frequency of the electromagnetic wave. This phenomenon is called dispersion. The position is determined based on the propagation time of the signal. Once the speed changes, the time and position are also different. The magnitude of the ionospheric effect is proportional to the number of free total electron content (TEC) electrons that fit in an unit cuboid from the receiver to the satellite. TEC is a function of many variable factors: time of day, solar activity, geographic location, and height of zenith satellites.

To eliminate the ionospheric error, dual-frequency receivers are used. In single-frequency receivers, the approximate correction is calculated and sent in the navigation message. To eliminate the positioning error caused by ionosphere when single-frequency GPS receivers are used, the Klobuchar model is used [56].

Due to the fact that satellite systems work on a huge scale, i.e., the satellites circulate in orbits and the distances between them and the receivers range from several to tens of thousands of kilometers, such a distance causes huge problems in the accuracy of position determination. Moreover, receivers and satellites must synchronize in real time, so any delay in the satellites’ basic atomic clocks can lead to significant errors. Since this research focuses on positioning and navigation in varying weather conditions, apart from the technical aspects of error generation, natural phenomenon from Earth’s atmosphere layers must also be taken into account. It seems that pressure, temperature, air humidity, or solar flares have a huge impact on the positioning accuracy. How and to what extent is analyzed in Section 3, but once the electromagnetic wave is delayed, the receiver cannot calculate the exact position of the satellite.

## 3. Satellite Signal Measurements in Various Weather Conditions

The measurements conducted and analyzed in this chapter are to answer the question how the changing weather conditions (in conjunction with Earth’s atmosphere) affect the accuracy of positioning in satellite systems, and what potential impact they may have on an autonomous machine movements based on GNSS technology usage. In theory, the ionosphere, dependent mainly on solar activity, has the greatest influence on the accuracy of position determination. For example, a solar storm can cause difficulties in the power supply or the operation of all electronics. Such extreme weather conditions were not encountered during the measurements. However, the pressure, temperature, or humidity varied, as well as solar activity, which was taken into account.

### 3.1. Measurements, Data Conversion Techniques

The measurement plan assumed 20 measurement days. Selected days were not consecutive, as different space and weather conditions were to be taken into account, but all of them occurred between October–December 2021. Once a day, GNSS data were collected using a satellite receiver. Every day, the necessary information about the external environment conditions was recorded, such as air temperature, humidity, wind speed, atmospheric pressure, and type of satellite systems used/satellites visible (see Section 3.2). Additionally, solar activity was recorded [57]. Measurements were conducted based on the absolute method, using one receiver and the satellite systems. The following steps (described in detail in the subsequent paragraphs of this chapter) of the data analysis procedure for each measurement were implemented:

1. Data aggregation, geographic coordinates determination at each measurement point;
2. Conversion of coordinates from World Geodetic System-84 (WGS-84) to flat Coordinate System 1992;
3. Calculation of the traveled trajectory in two directions: north–south, east–west, trend line, and its mathematical equation setting;
4. Determination of the arithmetic mean (in each direction), as well as the standard deviation;
5. Analysis, repetition of the procedure for subsequent measurements.

**Figure 1:**
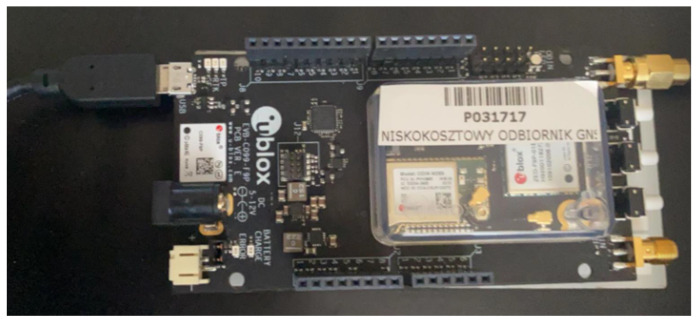
GNSS receiver used.

Ad 1. The receiver used was GPS UBLOX “P031717” receiver [58] (see Figure 1), which allows the recording of real-time trajectory traveled with the device. It is equipped with a GNSS module and may have an antenna attached. Every measurement was conducted on a straight road.

**Figure 2:**
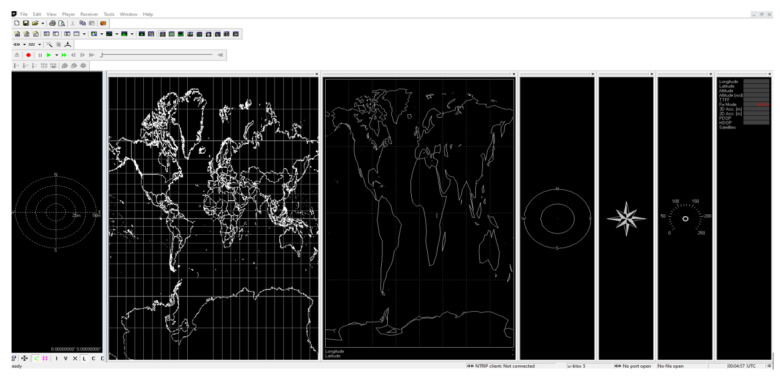
U-centre software interface.

The measurements were saved by the u-center software [58] in ubx format, an intuitive and easy-to-use application (see Figure 2). It contains many functions, such as a map of deviations, the ability to observe satellites’ position and their signals’ strength, as well as a compass or speedometer, for example. In addition, during real-time measurements it is possible to read the current latitude, longitude, altitude above the sea level, and DOP values.

After necessary conversions, it was possible to specify the number of satellites that were visible to the receiver during the measurements as well as from which satellite system they came from. Furthermore, signal strength, DOP factors, geographical coordinates, and altitude above sea level could also be determined. In order to analyze the measurement results, it was decided to determine the measurement uncertainty related to the course of the distance measured by the satellite receiver.

**Figure 3:**
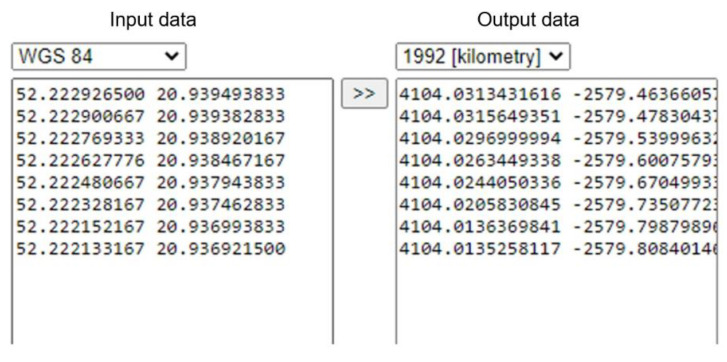
Geographical coordinates conversion.

Ad 2. Geographical coordinates of each measurement needed to be converted from World Geodetic System-84 (WGS-84) to flat Coordinate System 1992 in order to obtain the north–south and east–west directions (with assigned specific time). The coordinate conversion tool, available on website [59], was used to fulfill the task (see Figure 3). The input data represent geographic coordinates from the receiver, with the first number for longitude and the second for latitude. The output data show the x, y coordinates properly converted from the input data.

Ad 3. Thanks to the x, y data obtained (Table 1), it was possible to calculate the travelled trajectory in two directions: north–south, east–west. At the same time, the trend line and its mathematical equation was set.

**Figure 4:**
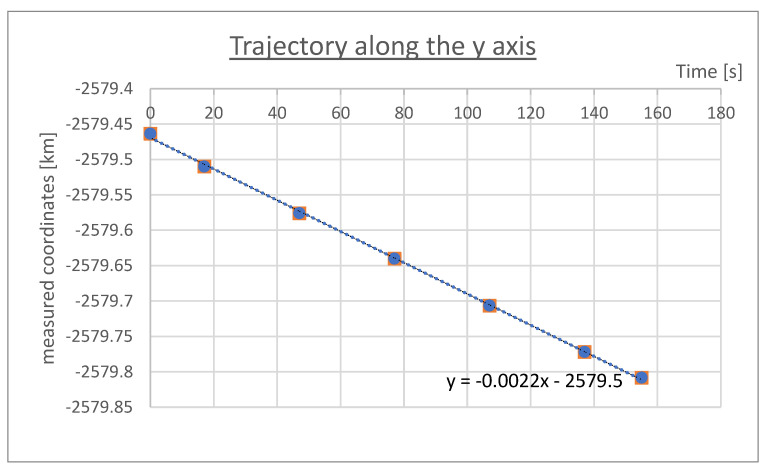
Geographical coordinates conversion, y axis.

**Figure 5:**
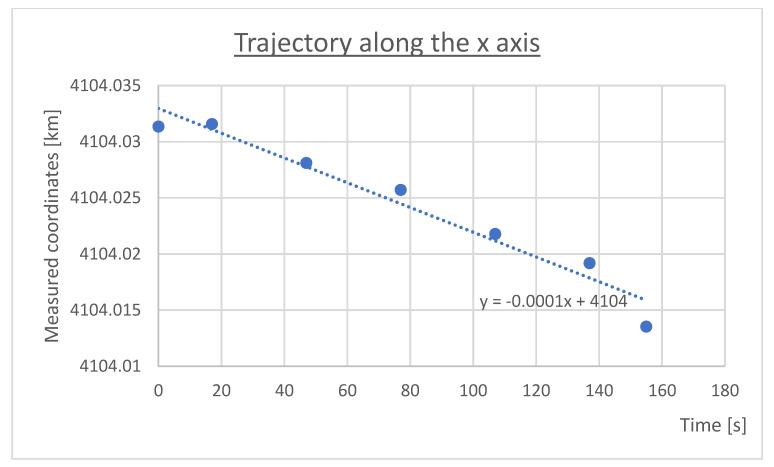
Geographical coordinates conversion, x axis.

Trend lines and their mathematical equations are presented on Figure 4—Equation (5) and Figure 5—Equation (6):

(5) y=−0.0022x−2579.5

(6) y=−0.0001x+4104

They allowed determination of the assumed positions, and calculation of coordinates x2 and y2, by substituting x with specified time (see Table 2).

Ad 4. The next step was to compare the obtained results with the measurement points read from the receiver. The arithmetic mean and the standard deviation were calculated to allow the comparison.

Arithmetic mean was calculated based on Equation (7) [60]:

(7) $$S_{a}=\bar{x}=\frac{\sum_{k=1}^{n}x_{k}}{n}$$

Standard deviation was calculated based on Equation (8) [60]:

(8) $$S=\sqrt{\frac{\sum_{i=1}^{n}{\left(x_{i}-\bar{x}\right)}^{2}}{n-1}}$$

where

$x_{i}$—individual measurements;

$\bar{x}$—average value of measurements;

n—number of measurements.

Calculation of standard deviations, both of the measured points and the results obtained from the trend line, made the comparison of those values and estimation of the measurement error possible (see Table 3).

Ad 5. The difference in the average deviations shows the average error in position determination by the GNSS receiver. In the above example: the error along Y axis is very small = 0.000074336 km ≈ 7.4 cm; the error along the X axis is equal to 0.00470937 km ≈ 4.7 m. The last measurement cannot be considered satisfactory.

The entire procedure was repeated for subsequent measurements on the following measurement days. The results obtained are shown in Table 4.

The values for measurement numbers six, eight, and fourteen are missing. It was impossible to read the data from the receiver. It is likely that no satellites were visible at the right moment or the measurement input was set incorrectly.

Generally, to sum this part up, it can be stated that position determination error varies. The lowest deviation value for Y axis is for measurement no 5: 0.00006751 km = 6.751 cm, and for X axis, measurement no 16: 0.00008695 km = 8.7 cm, which are perfect even for autonomous machines movements. On the other hand, the highest deviation for the Y axis is obtained for measurement no 18: 0.02027631 km = 20.28 m, and for X axis, measurement no 4: 0.03411303 km = 34.1 m, which is far from satisfactory! Did it depend on the weather conditions? Which component influenced the result the most?

### 3.2. Changing Weather Conditions during the Measurements

For each measurement, which means every day, the necessary information about the external environment conditions was recorded, such as air temperature, humidity, wind speed, atmospheric pressure, type of satellite systems used/satellites visible, and solar activity (Table 5). Weather conditions were independent of the receiver’s operator. To introduce even more variety, the measurements were conducted during various moments of a day: from morning until late at night, which was operator-dependent.

Temperature conditions varied from 3 °C in one evening to 15 °C, while the measurement was conducted at noon. Based on the measurement performed, it is difficult to draw a precise conclusion regarding temperature influence on positioning error.

The pressure varied from 996.36 hPa during one evening measurement, up to 1018 hPa on a forenoon. Humidity was the lowest during measurement no. 12, recorded as 55.4%. This was also the measurement during which the temperature reached the highest value. Humidity was the highest during measurement no. 8, reaching 97.13%, when the pressure also reached the highest value of all.

The wind speed was noted from 0.8 m/s up to 5.83 m/s. However, in the authors’ opinion, wind strength is dependent on the daytime moment (stronger in the forenoon and weaker in the evening).

During one half of the measurements, all satellite systems were available. That is positive. In six cases, satellites from only one satellite system (out of four) were visible to the receiver. For the three measurements (nos. 6, 8, and 14) where it was impossible to obtain required data, only one satellite system was available, and it was always Galileo.

What requires additional explanation to sum up the discussion on Table 5 is the last (but not least) column—solar flares. Solar flares are huge explosions on the sun that release energy, light, and high speed particles into space [61]. Adopted classification system divides solar flares according to their strength, based on their brightness in X-rays, measured in W/m^2^, and in the band from 0.1 up to 0.8 nm. The smallest ones are A-class (near background levels), then B, C, M, and X. Similar to the Richter scale for earthquakes, each letter represents a ten-fold increase in energy output (is 10 times stronger than the previous one). Therefore, an X is ten times an M and 100 times a C. Moreover, within each letter class there is a finer scale from 1 to 9. This means that, for example, an M2 flare is half of an M4 flare, and so on.

As written in [61] “C-class and smaller flares are too weak to noticeably affect Earth. M-class flares can cause brief radio blackouts at the poles and minor radiation storms that might endanger astronauts”. It may also affect the satellite navigation. The number of solar flares increases approximately every 11 years, which means that the upcoming solar maximum is likely to appear in 2024–2025.

Thanks to the continuous observation of the ionosphere and the sending of ionospheric corrections in navigation messages, during increased solar activity, the errors do not reach the maximum values to which this layer of the Earth’s atmosphere is able to contribute. Unfortunately, it was not noted whether the three M-class solar flares quoted had an Earth-directed component and whether the measurement place was facing the sun side, where the eruption took place. However, as a general effect on positioning accuracy may be noted, it was assumed they were directed to Earth. The influence of mentioned circumstances may be taken into account in the future development of research.

During measurements, significant M-class flares were noted for measurements nos. 11, 12, and 18. It can be seen, especially in measurement no. 18 (maximal error along Y axis), but for no. 12 as well (high positioning error around 16 m for both axis), that the positioning errors are larger, while the solar flares are stronger. On the other hand, the weakest solar flares identified are those for measurements nos. 4 and 5—B1.6. This also confirms the theory that the stronger the solar activity, the worse positioning (as such, a flare significantly affects the ionization of the ionosphere), and vice versa, as measurement no. 5 has the smallest positioning error along the Y axis. Tropospheric refraction does not have that much of an effect. It is usually omitted due to small errors and the difficulty of reducing the delays associated with it.

## 4. Conclusions

The main aim of this paper was to verify the influence of varying weather conditions on the accuracy of positional determination during navigation. As shown in the literature review, the issue of objects positioning accuracy (especially in transport applications) is extremely important, so solutions that increase the accuracy of position determination should be implemented. Both hardware and software solutions are used in this area. Moreover, the need to analyze this issue arises from the growing application of autonomous devices in everyday life. If they operate and move independently, precision of their navigation must be high, with centimeter-level accuracy, especially since they cannot use senses (to correct the position) in the same way as people.

To achieve the paper’s goal set, a short introduction of satellite systems and errors in position determination was presented. Then, satellite signal measurement in various weather conditions were conducted and discussion was attached. As a result of the carried-out research, it can be concluded that it is possible to achieved the expected accuracy. A large part of the position determination, based on conducted measurements, is satisfactory. Unfortunately, due to various types of errors, this does not apply to all of the measurements. The reason for this is, certainly, the constantly changing weather conditions, which affect the operation of satellite systems–receiver pair, as well as the reliability/visibility of satellite systems. Furthermore, to a large extent, the use of the absolute method of satellite signal measurements also contributed to the positioning errors. In the future, to increase the accuracy of position determination by GNSS systems, it is proposed to use a dual-frequency receiver that eliminates ionospheric refractions and an additional reference station with fixed geographic coordinates that would communicate with the receiver/autonomous machine. Such a solution would further increase accuracy and minimize the impact of errors generated by the environment. Development of telecommunication networks may positively influence navigation as well.

It must be also taken into account that the study was conducted for a fixed latitude. The effect of the ionosphere is critical in high-accuracy GNSS applications and it is highly dependent on the latitude. The effects are most intense in the equatorial region, moderate at high latitudes, and minimum at middle latitudes. To draw more reliable conclusions regarding the influence of space weather, measurements at different latitudes and during the entire solar cycle should be conducted, which is a field for future research.

The research carried out may be summed up in this way: environmental conditions, without doubt, affect the accuracy of position determination. The variability of environmental conditions is of great importance here and it is necessary to aim at solutions that reduce their impact on the accuracy of location. The ultimate (however, unwanted) solution is to rely on weather forecasts to capture extreme phenomenon. In case of solar activity influence, for example, forecasts can be useful to such an extent that on days with unfavorable weather conditions (solar flares of high classes), the autonomous machines can postpone their outdoor tasks in order to not be damaged due to positional inaccuracy.

## Figures and Tables

**Table 1:** x, y coordinates with assigned time.

Time [s]	y [km]	x [km]
0	−2579,463661	4104,031343
17	−2579,510050	4104,031565
47	−2579,576051	4104,028103
77	−2579,640251	4104,025706
107	−2579,706555	4104,021771
137	−2579,772036	4104,019176
155	−2579,808401	4104,013526

**Table 2:** x2, y2 coordinates with assigned time.

Time [s]	y [km]	x [km]	y2 [km]	x2 [km]
0	−2579,463661	4104,031343	−2579,5000	4091,687906
17	−2579,510050	4104,031565	−2579,5374	4091,687905
47	−2579,576051	4104,028103	−2579,6034	4091,687916
77	−2579,640251	4104,025706	−2579,6694	4091,687923
107	−2579,706555	4104,021771	−2579,7354	4091,687935
137	−2579,772036	4104,019176	−2579,8014	4091,687942
155	−2579,808401	4104,013526	−2579,8410	4091,687959

**Table 3:** Calculation of average deviations.

Time [s]	y [km]	x [km]	y2 [km]	x2 [km]
0	−2579,463661	4104,031343	−2579,5000	4091,687906
17	−2579,510050	4104,031565	−2579,5374	4091,687905
47	−2579,576051	4104,028103	−2579,6034	4091,687916
77	−2579,640251	4104,025706	−2579,6694	4091,687923
107	−2579,706555	4104,021771	−2579,7354	4091,687935
137	−2579,772036	4104,019176	−2579,8014	4091,687942
155	−2579,808401	4104,013526	−2579,8410	4091,687959
Arithmetic mean [km]	−2579,639572	4104,024456	−2579,669714	4091,687927
Average deviation [km]	0.092238622	0.00472354	0.092164286	0.0000141706205454284
**Deviation difference along Y axis [km]**	**0.000074336**		**deviation difference along X axis [km]**	**0.00470937**

**Table 4:** Differences in the standard deviations for all measurements.

Measurement Number	Deviation Difference along Y axis [km]	Deviation Difference along X axis [km]
1	**0.000074336**	**0.00470937**
2	0.00373305	0.01089750
3	0.00550258	0.01107862
4	0.00330949	0.03411303
5	0.00006751	0.00022016
6	-	-
7	0.01865231	0.00090735
8	-	-
9	0.00963509	0.00039513
10	0.00526347	0.00680266
11	0.00311555	0.00065142
12	0.01557693	0.01631348
13	0.01220545	0.00423828
14	-	-
15	0.00034146	0.00012886
16	0.00041352	0.00008695
17	0.00022089	0.00190523
18	0.02027631	0.00084854
19	0.00306734	0.00095147
20	0.00499201	0.00350703

**Table 5:** Weather conditions recorded during the measurements.

No	Daytime	Temp. [°C]	Pressure [hPa]	Humidity [%]	Wind [m/s]	Satellite Systems	Solar Flares [57]
1	Afternoon	12	997.6	58	3.61	All	B9.5 G2
2	Afternoon	11	1002.9	68	3.33	All	C1.3 Kp3+
3	Forenoon	8	1008.4	77	2.78	All	C2.2 Kp2+
4	Forenoon	10.2	1005.8	83.5	5.83	GPS	B1.6 Kp3
5	Evening	7	1007.3	92	2	GPS	B1.6 Kp3
6	Evening	8.5	1010.5	92.5	3.2	Galileo	B4.8 Kp2+
7	Evening	3	1013.5	96.8	2.3	All	B6.3 Kp2−
8	Forenoon	3.5	1018	97.13	2.1	Galileo	B6.2 Kp2−
9	Evening	3	1017.9	97	0.65	Glonass BeiDou	B6.2 Kp2−
10	Night	4	1008.6	91	2	All	C8.5 Kp2+
11	Evening	7.5	1005.3	85.1	1.8	Without BeiDou	M1.5 Kp1+
12	Noon	15	1005.2	55.4	3.8	Without Galileo	M1.5 Kp1+
13	Night	12	1001.6	70	3.5	Glonass	C3.9 Kp4-
14	Morning	10	1003.95	80	2.4	Galileo	C3.2 Kp4
15	Evening	6.1	996.36	97.1	1.8	All	C5.3 G2
16	Forenoon	9.2	1010.8	87.2	5.2	Without Galileo	C1.7 Kp4
17	Night	8.5	1009.5	82.3	4.8	All	C1.7 Kp4
18	Morning	5.2	1008.2	96.99	2.1	All	M1.5 Kp1+
19	Evening	15	999.2	95.6	0.8	All	C7.4 Kp4+
20	Evening	15	999.2	95.6	0.8	All	C7.4 Kp4+

## Data Availability

Not applicable.
